# Multi-species occupancy modeling suggests interspecific interaction among the three ungulate species

**DOI:** 10.1038/s41598-022-20953-7

**Published:** 2022-10-20

**Authors:** Hemant Singh, Amira Sharief, Bheem Dutt Joshi, Vineet Kumar, Tanoy Mukherjee, Kailash Chandra, Nitin Bhardwaj, Mukesh Thakur, Lalit Kumar Sharma

**Affiliations:** 1 Zoological Survey of India, New Alipore, Kolkata, 700053 West Bengal India; 2 Gurukul Kangri Vishwavidyalaya, Haridwar, 249404 Uttarakhand India; 3 Wildlife Institute of India, Dehradun, 248001 Uttarakhand India

**Keywords:** Animal behaviour, Behavioural ecology, Conservation biology, Ecological modelling

## Abstract

Species with sympatric distribution influence ecosystem dynamics and are impacted by the presence of other co-existing species. Assessing the coexistence and the role of interspecific interactions with the landscape variables is necessary to know the species co-occurrence in space. In the Indian Himalayan region, such studies are completely lacking due to limited efforts being made, mainly because of complex terrains and inaccessible landscape features. We used camera trapping and sign survey in a multi-species occupancy framework to understand the influence of environmental variables on occupancy and detection probability of species-specific and pair-wise interaction of the three ungulates in Uttarkashi. Our results concluded that individual species' occupancy probabilities were related both to the environmental variables and the presence or absence of other interacting species. Our top model showed evidence of interspecific interaction among species pairs, and the occupancy probability of species one varied in the presence or absence of another species. The overall activity patterns were similar among all the three species and were found active throughout the day. The activity overlap between sambar—barking deer (Dhat1 value = 0.85) was considerably higher than barking deer—goral (Dhat1 value = 0.78). The findings of the present study will be useful for the conservation and management of ungulates in the Indian Himalayan and adjoining regions.

## Introduction

In the Himalayas, the ungulates form the major prey base for the large predators and are good ecological indicators, more prone to anthropogenic disturbance and habitat quality^1,2^. Conservation strategies are primarily focused on the concept of multiple-species conservation in protecting endangered species and assessing biodiversity within entire communities^3,4^. Moreover, several studies demonstrated that ecological communities are composed of multiple interacting species, and species occurrence can be influenced by environmental factors and even the presence of other species^5,6^. Hence understanding those factors (environmental and species interactions) which influence the distribution of species is of fundamental interest in wildlife conservation and management. Multi-species occupancy modeling provides a method for assessing biodiversity while accounting for multiple sources of uncertainty, imperfect detections and sampling designs^7,8^. However, several studies highlighted that species occupancy probabilities are often influenced by many factors such as environmental factors and the presence or absence of interacting species, e.g. Predator’s presence for prey^9,10^, or habitat utilization may be impacted by the presence of other species^11^.

Among the ungulates, Barking deer (*Muntiacus vaginalis*), Himalayan goral (*Naemorhedus goral*), and Sambar deer (*Rusa unicolor)* have sympatric distribution in Himalayan regions and play an essential role in shaping ecosystems by influencing vegetation structure, forming a major prey base for carnivores and act as ecological indicators^12,13^. However, these species are threatened due to habitat loss, illegal poaching, climate change and interspecific competition^14–16^. Among the three studied species, the barking deer is widely distributed from eastern Pakistan, India, Nepal and up to south-east Asian countries, and mostly prefers high understory dense tropical and sub-tropical forest^17–19^. Barking deer also have a wide elevation distribution (100–3000 m) and are mostly found at the edge of forested habitats^20^. Whereas the Himalayan goral adapted to high elevation (900 to 2750 m) areas, it is a cliff-dwelling, solitary, sexually monomorphic mountain ungulate. Its distribution encompasses the Himalayan range of India, Nepal and Bhutan with densely forested areas to the sub-alpine scrub, alpine meadow and grassland^21,22^. The sambar is a large mesoherbivores deer native to South and South-East Asia and distributed in habitats with high tree and shrub densities^23^.

As per the IUCN Red List, barking deer is listed as ‘Least concern’^20^, sambar as ‘Vulnerable’^23^ and himalayan goral as ‘Near threatened’ species^24^. All three species are listed among the Schedule-III of the Indian Wild-Life (Protection) Act, 1972, and are seriously threatened because of illegal poaching. Since all three species are symmetrically distributed in Himalayan regions, such sympatric distribution of species may face exclusion^25^, avoidance^26^ or sometimes extinction due to increasing inter-specific competition^27^. Furthermore, limited studies on habitat use^28^, behaviour^29^, distributions^30–33^, genetics^22,23^ of these ungulates species is available but completely lacking evaluations on their occupancy and species interactions throughout their distribution ranges, including the Himalayan regions.

The Himalayan region is under tremendous pressure because of infrastructural development, climate change and other anthropogenic activities^34,35^. These events lead to rapid habitat loss, fragmentation, and population decline of various species. Therefore, understanding habitat association, occupancy and interspecies interaction are imperative for conservation and management. A better understanding of the ecology of the ungulate community may allow managers to more fully balance gains against losses when managing the diversity of wildlife^35,36^. Additionally, our research was motivated by the lack of broad-scale ungulate studies from the study area. This study explores the utility of camera trap surveys to understand species-specific and pair-wise interaction between three ungulate species using multi-species hierarchical models in the Uttarkashi district. These devices are helpful in areas with rugged topography or dense vegetation and, in recent years, have been used to study activity patterns, habitat use, density and occupancy of ungulates^37–39^. Modern cameras record the time of the photo, and researchers have used this to investigate diel activity patterns and compared activity patterns among species to see how overlapping patterns may relate to competition or predation^40–43^.

Our specific objectives were to evaluate species-specific responses to environmental variables and pair-wise interaction and the pattern of activity of the three ungulate species in Uttarkashi. We aimed to understand better how the environmental factors and interspecific interaction influence the occupancy of the three ungulates, which is vital for effective conservation and management implications.

## Results

### Occupancy of three sympatric species

**Figure 1:**
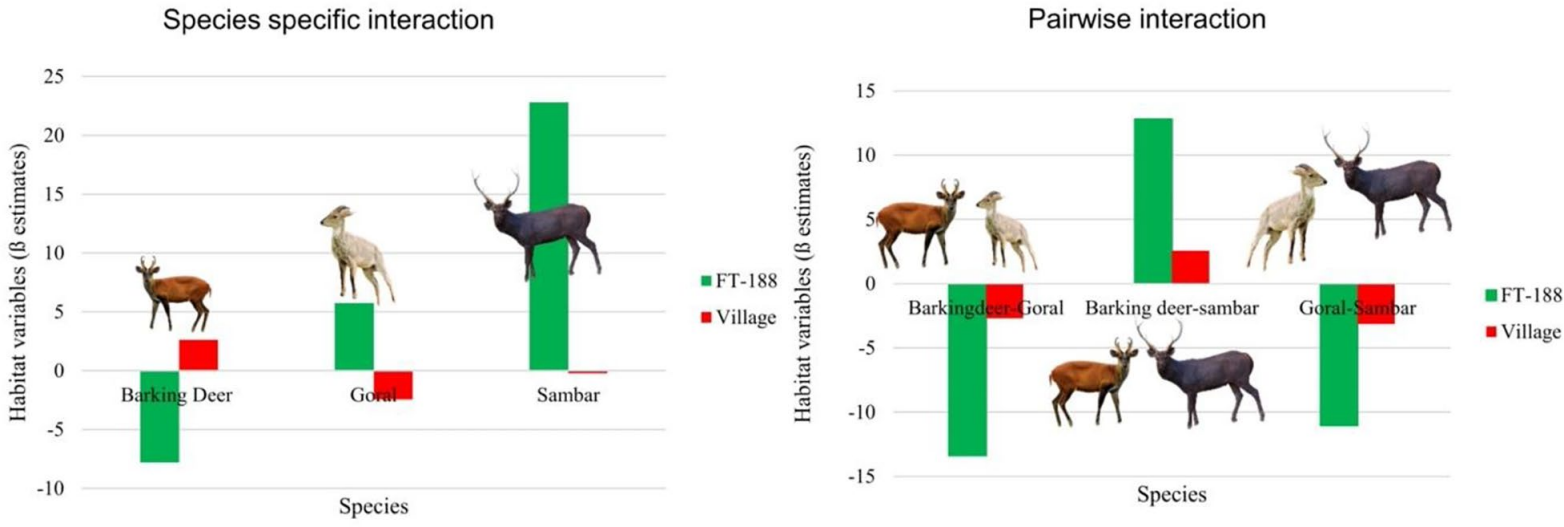
Species-specific and pair-wise interaction showing the influence of top model covariates (Green bars-forest type (West Himalayan Sub-alpine birch/fir Forest (FT 188) and Red bars distance to a village) on the occupancy of barking deer, goral and sambar in Uttarkashi. Microsoft office (MS version 2016) was used to create figures.

A sampling effort of 2819 camera trap nights across 62 camera sites was achieved in the Uttarkashi district. We obtained 30, 24, 9 detections of barking deer, goral and sambar across 62 sites and recorded 99, 92 and 82 indirect signs (faecal pellets) of each species. Our top model suggests evidence of interspecific dependence of the three species (*f*_12_, *f*_13_, (*f*_23_). The top model assumed the occupancy of the three species at different sites varied as a function of habitat variables ψ (FT 188 + Distance to the village) when detection probability was kept as constant p(.) (Table 1). This model suggests that the mean marginal occupancy probability of barking deer (*f*_1_) was positively influenced by distance to the village (β = 2.62 ± 1.13) and negatively influenced by FT 188 (West Himalayan sub-alpine birch/fir forest) (β = − 7.79 ± 0.00) (Table 2). We found that marginal occupancy of goral (*f*_2_) exhibits a positive relationship with variable FT 188 (β = 5.76 ± 0.00) and a negative relationship with variable distance to the village (β = − 2.43 ± 1.17). In contrast, marginal occupancy of sambar (*f*_3_) was positively influenced by FT 188 (β = 22.80 ± 0.00) and negatively influenced by distance to village (β = − 0.22 ± 0.59) (Table 2). Our top model results also showed evidence of interspecific interaction among species pairs (f12, f13, f23), which makes it very clear that the occupancy probability of species one varied in the presence or absence of another species (Fig. 1). We also found that the occupancy probability that two species occurred together varied as a function of FT 188 (Fig. 1). The influence of variable FT 188 on the co-occurrence of barking deer-goral (*f*_12_) and barking deer-sambar (*f*_13_) varied markedly. The relationship between the occurrence of barking deer (*f*1) and variable FT 188 varied markedly depending on whether goral (*f*2) and sambar (*f*3) were present. This model suggests that barking deer (*f*_1_) was more likely to occur together at sites where sambar (*f*_3_) was present (β = 12.80 ± 0.00), having FT 188 (West Himalayan sub-alpine birch/fir forest) as a variable function of occupancy, while both barking deer (*f*_1_) and goral (*f*_2_) (β = − 13.38 ± 0.00), and goral (*f*_2_) and sambar (*f*_3_) (β = − 11.05 ± 0.00), are less likely to occur together at sites with FT 188 (Fig. 1). Barking deer and sambar (*f*_13_) (β = 34.67 ± 163.39) were more likely to occur together than barking deer and goral (*f*_12_) (β = 16.50 ± 188.68) and goral and sambar (*f*_23_) (β = − 13.21 ± 188.68). This model also suggests barking deer–goral (*f*_12_) is negatively influenced by the variable (FT 188). The FT 188 showed negative influence on co-occurrence of barking deer-goral (*f*_12_) (β = − 13.38 ± 0.00) and goral-sambar (*f*_23_) (β = − 11.05 ± 0.00), while showed positive influence on occupancy of barking deer-sambar (*f*_13_) (β = 12.80 ± 0.00). The model also showed that the probability of two species occupying the same site as a function of covariates provided insight into factors driving marginal occupancy probabilities that might not have been evident otherwise. Our results concluded that individual species' occupancy probabilities were related both to environmental variables and the presence or absence of other interacting species (Fig. 1).

**Table 1:** Summary of top five models selected for multi-species occupancy of three ungulates in Uttarkashi Uttarakhand.

S.N.	Model	AICc	Delta AICc	AICc weights	Modellikelihood	K
1	ψ (FT188 + vill), p(.)	920.95	0	0.76	1	10
2	ψ (FT188 + vill), p(slope)	924.58	3.63	0.12	0.16	16
3	ψ (FT188 + vill),p(vill)	925.15	4.20	0.09	0.12	16
4	ψ (FT188 + FT203),p(slope)	928.28	7.33	0.01	0.02	12
5	ψi(FT188),p(slope)	933.12	12.17	0.000	0.001	9

**Table 2:** Top five models with beta values showing influence of different site covariates, species specific interaction and pair-wise interaction on occupancy and detectability of three ungulate species.

Interaction	Species	Model	ψ(FT188)	ψ (vill)	p(slope)	ψ(FT-203)	p(vill)
F1	Barking deer	ψ(FT188 + vill), p(.)two way	− 7.79 ± 0.00	2.62 ± 1.13	–	–	–
		ψ(FT188 + vill), p(slope)two way	− 10.03 ± 0.00	–	0.18 ± 0.06	0.006 ± 1.54	–
		ψ(FT188 + vill),p(vill)	9.05 ± 101.9	0.41 ± 1.32	–	–	0.18 ± 0.13
		ψ(FT188 + FT203),p(slo)	23.39 ± 967.3	–	0.19 ± 0.06	− 2.81 ± 1.51	–
		ψ(FT188),p(slope)	20.28 ± 102.1	–	0.18 ± 0.06	–	–
F2	Goral	ψ(FT188 + vill), p(.)two way	5.76 ± 0.00	− 2.43 ± 1.77	–	–	–
		ψ(FT188 + vill), p(slope)two way	− 1.31 ± 222.78	− 2.47 ± 1.17	− 1.42 ± 0.35	–	–
		ψ(FT188 + vill),p(vill)	2.69 ± 0.00	− 2.71 ± 1.51	–	–	0.54 ± 0.21
		ψ(FT188 + FT203),p(slo)	− 20.43 ± 624.63	–	0.032 ± 0.08	2.09 ± 1.34	–
		ψ(FT188),p(slope)	− 20.25 ± 526.21	–	0.05 ± 0.08	–	–
F3	Sambar	ψ(FT188 + vill), p(.)two way	22.80 ± 0.00	− 0.22 ± 0.59	–	–	–
		ψ(FT188 + vill), p(slope)two way	15.60 ± 326.90	0.45 ± 181.32	0.22 ± 0.10	–	–
		ψ(FT188 + vill),p(vill)	− 3.79 ± 0.00	2.09 ± 212.13	–	–	0.41 ± 0.14
		ψ(FT188 + FT203),p(slo)	26.35 ± 108.02	–	0.22 ± 0.10	− 0.28 ± 0.70	–
		ψ(FT188),p(slope)	19.43 ± 593.57	–	0.22 ± 0.10	–	–
F12	Barking deer-goral	ψ(FT188 + vill), p(.)two way	− 13.38 ± 0.00	–	–	–	–
		ψ(FT188 + vill), p(slope)two way	− 20.50 ± 0.00	− 2.68 ± 2.15	0.18 ± 0.06	–	–
		ψ(FT188 + vill),p(vill)	− 14.28 ± 0.00	− 2.65 ± 245.63	–	–	0.18 ± 0.13
		ψ(FT188 + FT203),p(slo)	–	–	–	–	–
		ψ(FT188),p(slope)	–	–	–	–	–
F13	Barking deer-sambar	ψ(FT188 + vill), p(.)two way	12.80 ± 0.00	–	–	–	–
		ψ(FT188 + vill), p(slope)two way	12.76 ± 289.62	2.57 ± 189.34	0.09 ± 0.08	–	–
		ψ(FT188 + vill),p(vill)	19.13 ± 0.00	− 0.03 ± 245.03	–	–	0.54 ± 0.21
		ψ(FT188 + FT203),p(slo)	–	–	–	–	–
		ψ(FT188),p(slope)	–	–	–	–	–
F23	Goral-sambar	ψ(FT188 + vill), p(.)two way	− 11.05 ± 0.00	–	–	–	–
		ψ(FT188 + vill), p(slope)two way	− 15.88 ± 0.00	− 3.13 ± 3.13	0.22 ± 0.10	–	–
		Psi(FT188 + vill),p(vill)	− 23.01 ± 0.00	2.18 ± 245.64	–	–	0.41 ± 0.14
		ψ(FT188 + FT203),p(slope)	–	–	–	–	–
		ψ(FT188),p(slope)	–	–	–	–	–

### Activity pattern

**Figure 2:**
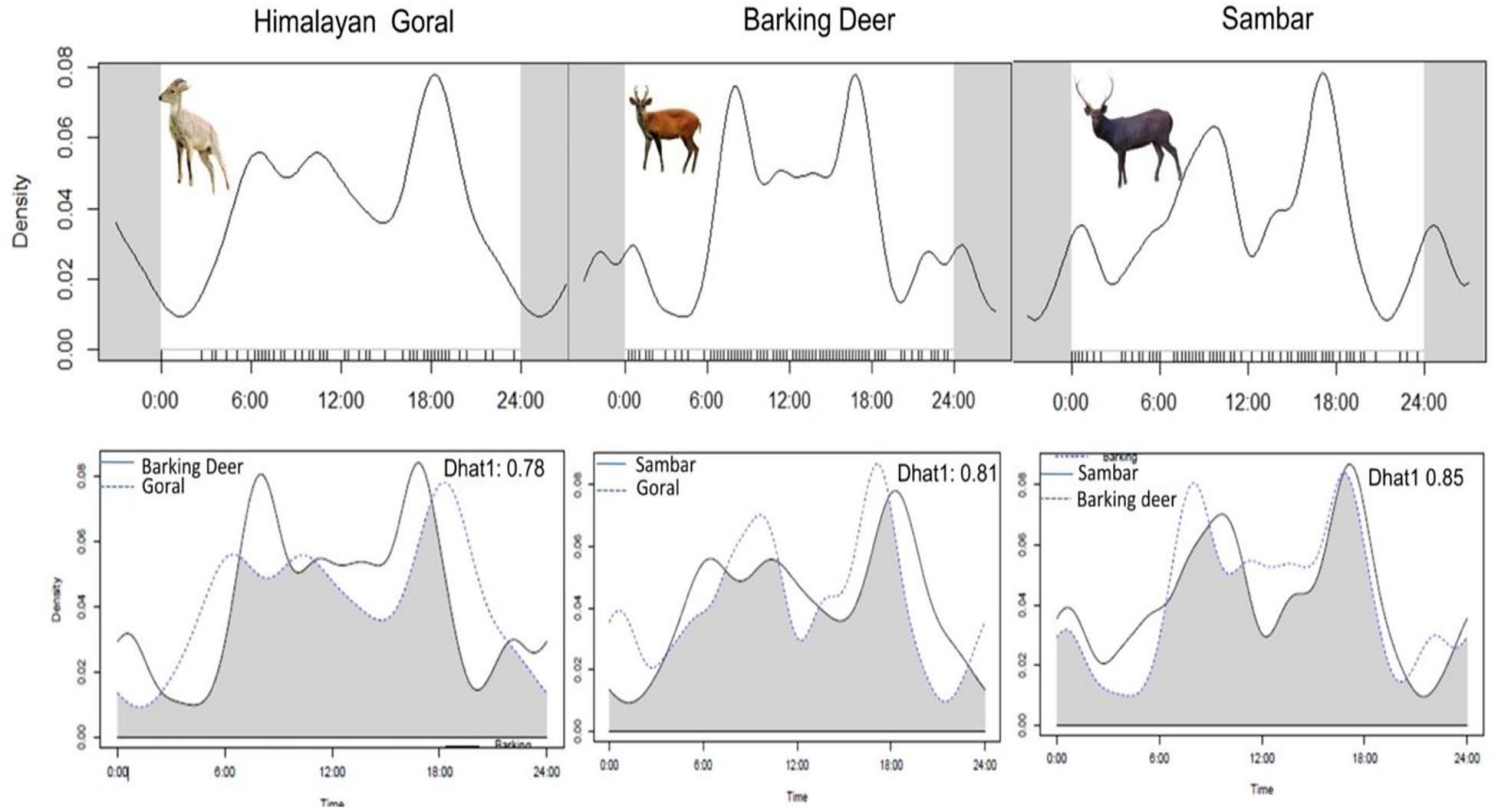
Temporal activity pattern of three ungulate species (goral, barking deer and sambar—top row) and overlapping activity pattern of barking deer-goral, Goral-sambar, and sambar-barking deer (Bottom row). Overlap package of R-Software (R.3.4.3, https://www.R-project.org) was used to create the figure.

The overall activity patterns were similar among all the three ungulate species. All the species were active throughout the day, with decreased activity between early morning, afternoon and late evening hours. Goral was active throughout the day with decreased activity in the early morning and evening hours (Fig. 2). In contrast, both barking deer and sambar were more active in the late morning and early evening hours (Fig. 2). The overlap of activity between sambar—barking deer (Dhat1 value = 0.85) was considerably higher than barking deer—goral (Dhat1 value = 0.78), and to a very lesser extent than goral—Sambar (Dhat1 = 0.81) (Fig. 2).

## Discussion

For identifying an area of conservation importance, it is vital to know the spatial distribution of the good quality habitat of the species. The distribution of species in space is influenced by various environmental factors and interactions with other species. Studies have modeled the occupancy of ungulates using the occupancy models^15^. The recent improvement in analytics has enhanced the capability of ecologists to model the detection/non-detection data for multiple species while incorporating imperfect detection in the data^6,44–46^. In this study, we evaluated the influence of environmental variables on occupancy and detection probability of species-specific (*f*1, *f*2, *f*3) and pair-wise interaction (*f*12, *f*13, *f*23) of three ungulate species in Uttarkashi. The top model assumed the occupancy of the all thethree species at different sites varied as a function of habitat variables ψ (FT 188 + Distance to village) when detection probability was kept as constant p(.) (Table 1). This model suggests that the mean marginal occupancy probability of barking deer (*f*1) was positively influenced by distance to the village and negatively influenced by FT 188 (West Himalayan sub-alpine birch/fir forest) (Table 2). We found that marginal occupancy of goral (*f*2) exhibits a positive relationship with variable FT 188 and a negative relationship with variable distance to the village. In contrast, marginal occupancy of sambar (*f*3) was positively influenced by FT 188 and negatively influenced by distance to the village (Table 2). Our top model results also showed evidence of interspecific interaction among species pairs (f12, f13, f23), which makes it very clear that the occupancy probability of species one varied in the presence or absence of another species (Fig. 1). Our study showed that the two species occupying the same site is a function of covariates that provides insight into factors driving marginal occupancy probabilities that might not have been evident otherwise^6^. Our results corroborate with the findings of other study^6^ that the occupancy probabilities of individual species are directly related to the habitat variables as well as the presence or absence of other interacting species.

This is the first attempt to model multi-species occupancy of ungulates from Uttarkashi, Uttarakhand, and the entire Himalayan region. We found evidence for pair-wise interactions (*f*12, *f*13, *f*23) among three ungulate species that varied along with environmental variables in the present study. Nonetheless, we observed the anticipated negative association of FT 188 with pair-wise interaction of *f*_12_ (barking deer-goral) and *f*_23_ (goral-sambar). However, pair-wise interaction showed; that occupancy of *f*_13_ (barking deer-sambar) was positively influenced by FT 188. Of the three species, goral (*f*_2_) showed the broadest elevation range covering up to 3600 m. Other studies on the ecology and distribution of goat-antelopes also indicated a broad elevation range for goral^2,47–49^ in western Himalayas. These studies indicated that goral could be found from very low elevation (500 m) to the tree line (4000 m). Since FT 188 is negatively influencing the pair-wise interaction of barking deer-goral and sambar-goral, we suggest that this might be because of resource partitioning, which leads the species to occupy different habitats. Goral inhabits steep mountainous areas and sometimes use forests near cliffs but primarily stays within rugged rocky terrain covering a broad elevation range. Whereas sambar and barking deer are forest dwellers, sambar (*f*_3_) prefers hilly areas with high tree density and showed the least affinity towards human settlement, while barking deer prefers plain areas^30,50,51^.

The model provides the probability of pair-wise interaction that two species occur together as a function of covariates. FT 188 positively influenced the marginal occupancy of goral and sambar and negatively influenced the marginal occupancy of barking deer**.** Since species-specific occupancy of barking deer (*f*_1_) was negatively influenced by FT 188 whereas FT 188 positively influenced the pair-wise interaction of barking deer-sambar (*f*_13_) which suggested barking deer (*f*_1_) was more likely to occur together at sites where sambar was present. Many species occur in sympatry where intraguild competition is frequent. Further, intraguild competition is an important determinant in structuring ecological communities, as it can lead to spatial or temporal segregation among species. Since we found evidences that the interaction between barking deer and sambar in FT 188 is positive, we suggest that the dietary overlap and resource competition of the two species might be small enough to allow coexistence.

Moreover, our findings were similar to that of^52^, which suggests barking deer also showed a positive association towards distance to the village, although barking deer (*f*_1_) prefers dense forest with high understory^53^. However, barking deer (*f*_1_) are also reported to occupy degraded tracts of forest ranges near human settlements. Their inability to live on rugged steep mountain slopes allows them to occupy forested habitats close to human habitations^53^. At the same time, goral (*f*_2_) and sambar (*f*_3_) showed a negative association toward distance to the village. Both the species are shy and mostly solitary thus, avoidance of villages was quite normal, similar to the results of^54^. The goral (*f*_2_) occupancy was associated with a wide variety of habitats throughout the mountain range^28^, and predominately, it is adapted to steep slopes with rugged mountain terrain^50,55,56^.

The activity pattern results suggested that the goral (*f*_2_) was active throughout the day with decreased activity in the early morning and evening hours (Fig. 2). In contrast, both the barking deer and sambar were more active in the late morning and early evening hours (Fig. 2). The goral was found active during the day due to its cleft dwelling nature, where it escapes from predators and the human presence. The overlap of activity between sambar—barking deer was considerably higher than barking deer—goral, and to a lesser extent, than goral—sambar, possibly due to different habitat preferences (Fig. 2). There is much similarity between all the three species; however, ecological separation nevertheless, the complete absence of sambar above 3200 m and avoidance of sambar to coniferous forests indicates a certain degree of ecological separation between the three species. Goral (*f*_2_) is probably associated with more tree cover in winter because of their vulnerability to predation and food availability during heavy snow conditions. Such factors could influence the increased use of tree cover by the goral in summer as the need to find shelter during the monsoon or the need to secure cover for parturition. The biotic and abiotic conditions are essential for understanding the interspecific interactions as they determine the site utilization by the species. Hence, researchers have recognized their imperatives as covariates for modelling the space use, habitat selection^57^, and distribution^58^ assessment of a species. Accounting for the interspecific interactions may be necessary when predicting future distributions, predominantly in understanding the impacts of global warming and climate change on the distribution of species and communities. Hence, the present multi-species occupancy model provides a strategy for understanding the interspecific interactions for imperfectly detected species.

Moreover, it may help in evaluating how the interspecific interactions shape habitat selection and species distributions. Among the three ungulate species studied, sambar and goral are threatened according to the IUCN category. Habitat loss, anthropogenic pressure, and wildlife hunting pose a formidable challenge for conserving wildlife^58^. The long-term survival of wildlife depends on sufficiently large areas of suitable habitat and decreasing anthropogenic pressure. The present findings may be helpful for the better conservation and management of the mountain ungulates in the Indian Himalayan Region and other adjoining areas. Hence, we propose that areas supporting suitable habitats for all three mountain ungulates should seldom have anthropogenic activity for proper management and conservation of the species given the influence of FT 188 on the occupancy of the three species, we would recommend plantation and assisted natural regeneration of the local plant species in suitable gap areas for supporting the ungulates in the landscape.

Complex terrain, inaccessibility and heavy snowfall are the limitations which make it difficult to study the species in the landscape. Due to weather conditions, heavy rainfall, landslides and snowfall during winters, we were not able to do sampling throughout the year, which is the major limitation of the study. Inferences have been made on the basis of short sampling effort, however, long-term study with intensive sampling is required for proper conservation and management of the ungulates. Though our data was collected relatively from a small area, information on the environmental factors governing the distribution of the ungulates would establish the baseline information on the distribution of ungulates in the landscape.

## Material and methods

### Study area

**Figure 3:**
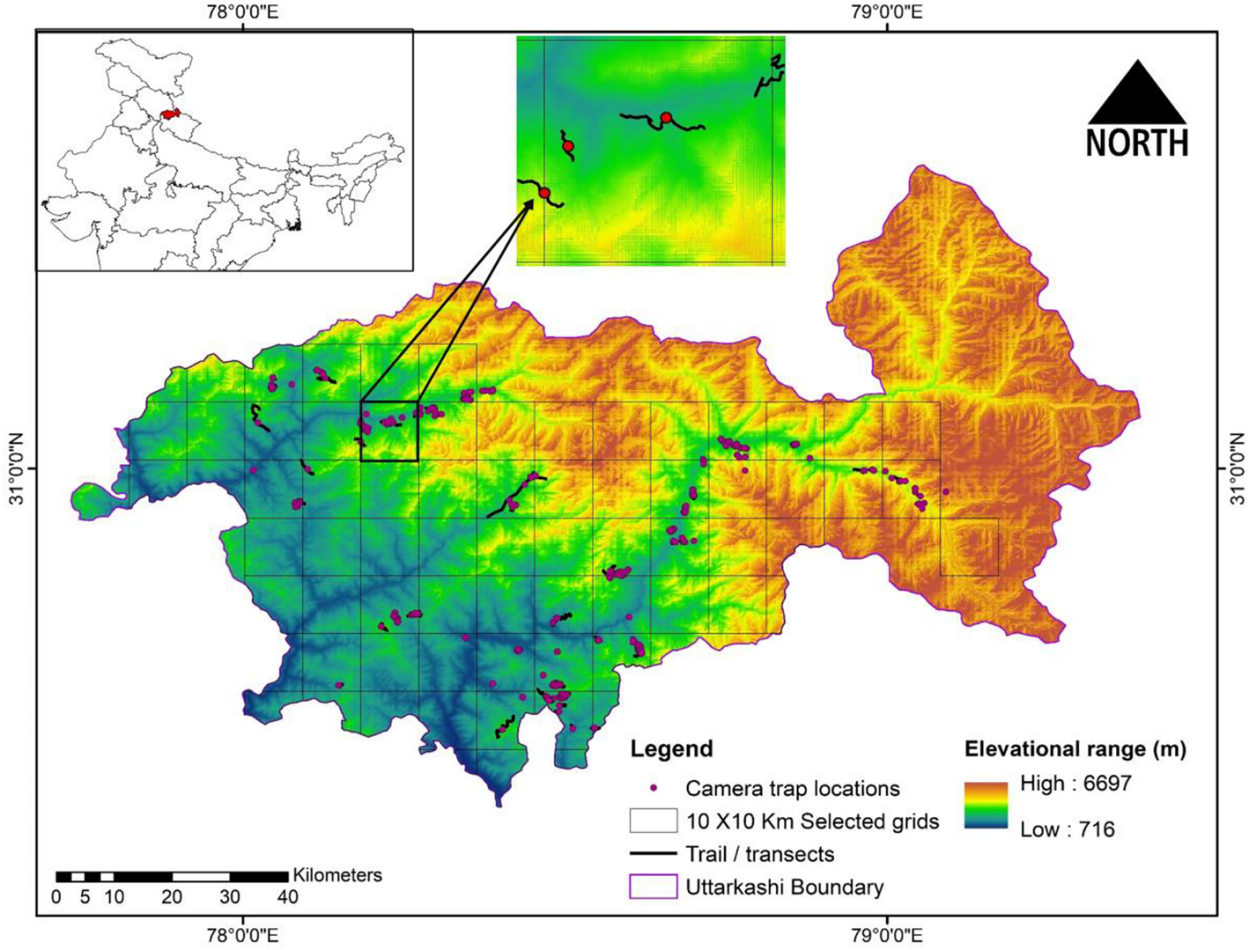
Map of the study area Uttarkashi, Uttarakhand. ArcGIS 10.6 (ESRI, Redlands, CA) was used to create the map. (Map created using ArcGIS 10.6; http://www.esri.com).

The present study was conducted in Uttarkashi district, Uttarakhand, located between 38° 28′ to 31°28′ N latitude and 77°49′ to 79°25′ E longitude with an area of about 8016 km^2^, covering primarily hilly terrain with an altitudinal range of 715–6717 m (Fig. 3). The terrain is mountainous, consisting of undulating hill ranges and narrow valleys with temperate climatic conditions. The district lies in the upper catchment of two major rivers of India, viz., the Ganges (Bhagirathi towards upstream) and the Yamuna. The major vegetation types of the study area are Himalayan moist temperate forest, sub-alpine forest and alpine scrub^59^. The Uttarkashi district forests are managed under three Forest Divisions viz., (i) Uttarkashi Forest Division (ii) Upper Yamuna Badkot Forest Division and (iii) Tons Forest Division) with two Protected Areas (PAs) (i) Gangotri National Park and (ii) Govind Pashu Vihar National Park. The forested habitats of the study landscape are home to top conservation priority species, including Asiatic Black bear (*Ursus thibetanus*), Musk deer (*Moschus *spp.), Common leopard (*Panthera pardus*), Himalayan brown bear (*Ursus arctos isabellinus*) and Western Tragopan (*Tragopan melanocephalus*), Himalayan monal (*Lophophorus impejanus*). The study was conducted after a study permit issued by the Chief Wildlife Warden, Forest Department, Uttarakhand government, vide letter no. 848/5-6 dated 31/08/2019, we have not handled the species for doing research. Instead, remote camera traps have been used for collecting the data with the permission of the Chief Wildlife Warden, Government of Uttarakhand. Further, informed consent was taken before interviewing the local communities. The data was collected according to the institutional guidelines and approved by the Research Advisory and Monitoring Committee of the Zoological Survey of India.

### Sampling protocol

The basic sampling protocol and assumptions for multi-species occupancy modelling are identical to the single-species case^7^. Briefly, a set of 62 intensive sites, were randomly selected, and each site *i* was surveyed *j* times. During each survey, detection/non-detection of S focal species was recorded. Additionally, direct or indirect evidences of species presence from the different areas were also recorded.

### Data collection

The complete study area was divided into 10 × 10 km grids, consisting of n = 60 grids. Based on the reconnaissance survey, out of these 60 grids, we selected 25 girds that were accessible to conduct the survey and have the species presence. Further, these grids were divided into 2 × 2 km grids to maximize our effort so that all logistically accessible grids could be covered, and we conducted intensive sampling in N = 62 grids after excluding the grids with human settlements. T The field surveys were conducted during 2018–2019, and a team of researchers systematically visited selected grids to collect data on the detection/non-detection of these ungulates. A total of 62 camera traps were deployed in selected grids, and 650 km were traversed, accounting for N = 54 trails in these sampled grids. These camera traps were visited once in every fifteen days for replacing the batteries as well as documenting the presence of the species through the sign surveys. The ultra-compact SPYPOINT FORCE-11D trail camera (SPYPOINT, GG Telecom, Canada, QC) and Browning trail camera (Defender 850, 20 MP, Prometheus Group, LLC Birmingham, Alabama, https://browningtrailcameras.com) camera traps were used to detect the presence/absence of ungulate species. The cameras were mounted 40–60 cm above ground on natural trails without lures.

### Data exploration

While deploying camera traps, we also noted habitat variables through on-site observation such as distance to the village and human disturbance. We tested site covariates for collinearity and discarded one of a pair if the Pearson’s correlation was greater than 0.7^60^. Hence, we assumed each of the site covariates could influence the occupancy and detectability of these ungulates.

### Covariates

We hypothesized that habitat variables may influence these ungulates' occupancy and detection probability. A total of 21 variables were extracted either from the field or using the ArcGIS v. 10.6 software (ESRI, Redlands, CA), and only 14 were retained after collinearity testing^60^ (Table 3). These covariates were classified into the following categories (Topographic variables, Habitat variables and anthropogenic variables). The topographic variables (elevation, slope and aspect) were generated using 30× resolution SRTM (Shuttle Radar Topography Mission) image downloaded from EarthExplorer (https://earthexplorer.usgs.gov/). The habitat/ land cover classification was carried out using Landsat 8 satellite imagery (Spatial resolution = 30 m) downloaded from Global Land Cover Facility by following the methodology suggested by^61^ using the ArcGIS v. 10.6 software (ESRI, Redlands, CA). The study area was classified into nine Land use/land cover (LULC) classes viz., West Himalayan Sub-alpine birch/fir Forest (FT 188), West Himalayan upper oak/fir forest (FT 162), West Himalayan Dry juniper forest (FT 180), Ban oak forest (FT 152), Moist Deodar Forest (FT 155), Western mixed coniferous forest (FT 156), Moist temperate Deciduous Forest (FT 157) which were used for further analysis considering their importance to species ecology and behavior^60^. The values for all the covariates were extracted at 30 m resolution, and a single value per site was obtained by averaging all the pixel values within each sampling site (camera trap locations).

**Table 3:** Habitat variables used for multi species occupancy analysis of three ungulate species in Uttarkashi, Uttarakhand.

S. no.	Variable	Code	Data	Source
**LULC/land use land cover type**				
1	West Himalayan upper oak/fir forest	FT 162	LANDSAT 8	USGS
2	Ban oak forest	FT 152		
3	Moist deodar forest	FT 155		
4	Moist temperate deciduous forest	FT 157		
5	Western mixed coniferous forest	FT 156		
6	West Himalayan Dry juniper forest	FT 180		
7	West Himalayan sub alpine birch/fir forest	FT 188		
8	Non forested area	FT 203		
9	Distance to village	vill	Calculated using log Euclidean distance (ArcGIS 10.6)	LULC map
10	Distance to water	DW		
11	Distance to road	DR		
**Topographic variables**				
12	Aspect	ASP	SRTM	USGS
13	Slope	SLP		
14	Elevation	ELE		

### Occupancy modelling framework

We used multi-species occupancy modelling^62^ of barking deer, goral and sambar to estimate the probability of the species (*s)* occurred within the area (*i*) sampled during our survey period (*j*), for accounting the imperfect detection of the species^8^. Distinguishing the true presence/absence of a species from detection/non-detection (i.e., species present and captured or species present but not captured) requires spatially or temporally replicated data. We used camera stations to record the presence/absence of species along with sign survey in all the studied grids. The camera traps were placed along the trail/transects in the studied grids hence each grid needs to be visited once in every fifteen days to check the camera traps as well as to document the presence of the studied species. Therefore, we treated 15 trap nights as one sampling occasion at a particular camera station resulting in ~ 7 sampling occasions per camera station.Our aim was to record the presence/ absence of the species at a particular gird hence we incorporated sign survey data if the species was not detected in camera station but recorded through sign survey. We pooled the presence/absence data in a single sheet of each species following^6^ and fitted occupancy and detectability models using programme Mark^63,64^. We model the species (*s*) presence (*y*_*sij*_ = 1) and absence (*y*_*sij*_ = 0) at site *i* during survey *j,* and the sampling protocol was identical to single species case^65^, where the Bernoulli random variable was conditional on the presence of species s (Z_*s*_ = 1) following^6^

$${\text{y}}_{sij} \sim {\text{ Bernoulli}}\left( {{\text{p}}_{sij} {\text{z}}_{si} } \right),$$

where P*sij* represents the probability of detecting species *S* during replicate survey *j* at site *i* and Z*si* = presence or absence of species *s* at site *i*.

Furthermore, we model the latent occupancy state of species *s* at site *i* as a multivariate Bernoulli random variable:

$${\text{Z}}_{i} \sim {\text{MVB}}\left( {\uppsi _{i} } \right)$$

where Z_*i*_ = {Z_1*i*_, Z_2*i*_….., Z_*Si*_} is an S-dimensional vector of 1’s and 0’s denoting the latent occupancy state of all *S* species and (ψ_*i*_) is a 2^S^-dimensional vector denoting the probability of all possible sequences of 1’s and 0’s Z_*i*_ can attain such that ∑ **ψ**_***i***_ = 1 with corresponding probability mass function (PMF) adopted from^6,64^.

$$f\left( {{\text{Z}}_{i} } \right) = {\text{ exp}}\left( {\left( {{\text{Z}}_{i} {\text{log}}(\uppsi_{{\text{i}}} {1}/\uppsi_{{\text{i}}} 0} \right) \, + {\text{ log}}\left( {\uppsi_{{\text{i}}} 0} \right)} \right).$$

The quantity *f* = log (ψ_*i*_1/ψ_*i*_0), is the log odds species S occupies a site often referred to as a ‘natural parameter’.

Since we are modeling three ungulate species (S = 3), 2^S^ = 2^3^ the possible encounter histories included in the dataset were eight, if neither of the two species were detected the value of ‘00’ was assigned; similarly ‘01’ indicates detection of species 1; ‘02’ indicates detection of species 2; ‘03’ indicates detection of both the species; ‘04’ indicates detection of species 3; ‘05’ indicates detection of species 1 and species 3; ‘06’ indicates detection of species 2 and species 3 and ‘07’ indicates detection of all the three species. We modelled constant occupancy and detection probability for each of the three species. Hence, we specified 6 *f* and p parameters, an intercept (β) for each of one-way *f* parameter and detection parameter p following^64^.

$$f_{{1}}=\upbeta_{{{1},}} \;\;{\text{p}}=\upbeta_{{4}}$$

$$f_{{2}} = \upbeta_{{{2},}} \;{\text{p }} = \, \upbeta 5$$

$$f_{{3}} = \, \upbeta_{{{3},}}\; {\text{p }} = \, \upbeta_{{6}}$$

We fit a set of models including the detection probability as a constant, p(.), and variable function to occupancy ψ(covariate) for site-specific covariates and models include occupancy as constant ψ(.) and variable function of the detection p(covariates) for the respective site covariates.

As we have assumed the independence among all three species, the model shows marginal occupancy probabilities of species 1, species 2 and species 3 varies as a function of environmental variables. We incorporated site-level characteristics affecting species-specific occurrence (*f*1: occupancy of species 1, *f*2: occupancy of species 2, & *f*3: occupancy of species 3) and detection probabilities using a generalized linear modelling approach^42^. This requires 9 parameters: an intercept (β_1,_ β_3,_ β_5_) and slope (β_2,_ β_4,_ β_6_) coefficient for each 1-way *f* parameter *f*_1_, *f*_2_, *f*_3_ and an intercept parameter for each detection parameter (β_7,_ β_8,_ β_9_). Below mentioned is the model for 1-way *f* parameters.

$$f_{{1}} = \, \upbeta_{{{1 } + }} \upbeta_{{2}} \left( {{\text{Covariate}}} \right),\;\;{\text{ p }} = \, \upbeta_{{7}}$$

$$f_{{2}} = \, \upbeta_{{{3 } + }} \upbeta_{{4}} \left( {{\text{Covariate}}} \right),\;\;{\text{ p}} = \, \upbeta_{{8}}$$

$$f_{{3}} = \, \upbeta_{{5}} + \, \upbeta_{{6}} \left( {{\text{Covariate}}} \right),\;\;{\text{ p }} = \, \upbeta_{{9}} .$$

All covariates were standardized before model fitting. We fitted the most complex model to each species and considered all possible combinations of covariates using the logit link function. Our rationale for including these variables in the occupancy and detectability component of the model was that we expected these variables to influence the occupancy and detectability of the study species.

Since multi-species occupancy simultaneously model environmental variables, & interspecific interactions. Further it also allows to understand the influence of environmental variables on one species occupancy, in the presence or absence of other sympatric species^64^. Hence, we also modeled two species occur together as a function of covariates. We examined how the variables of each camera site influenced the pair-wise interaction of the three ungulate species. This model assumes that the conditional probability of one species varies in the presence or absence of other species. We assumed *f*123: co-occurrence of species 1, species 2 & species 3 = 0, hence we did not include higher-order interactions in any of our models, we assumed the conditional probability of 3 species occurred together was purely a function of species-specific (*f*1, *f*2, *f*3) and pair-wise interaction (*f*12: co-occurrence of species1 & species 2, *f*13: co-occurrence of species 1 & species 3, *f*23: co-occurrence of species 2 & species 3) parameters. We modeled pair-wise interaction of species varies as a function of environmental variables keeping detection probability constant. Hence, we specified 15 *f* and *p* parameters, an intercept and slope coefficient for each of the one-way (*f*1, *f*2, *f*3) and the two-way *f* parameters (*f*12, *f*13, and *f*23); as well as an intercept parameter for each of the detection models. The model equation below implies for 2-way *f* parameters:

$$f_{{{12}}} = \, \upbeta_{{{7 } + }} \upbeta_{{8}} \left( {{\text{Covariate}}} \right),\;\;{\text{ p }} = \, \upbeta_{{{13}}}$$

$$f_{{{13}}} = \, \upbeta_{{{9 } + }} \upbeta_{{{1}0}} \left( {{\text{Covariate}}} \right),\;\;{\text{ p }} = \, \upbeta_{{{14}}}$$

$$f_{{{23}}} = \, \upbeta_{{{11 } + }} \upbeta_{{{12}}} \left( {{\text{Covariate}}} \right),\;\;{\text{ p }} = \, \upbeta_{{{15}}} .$$

We also fitted models including co-occurrence and detection probability of a species varies as a function of environmental variables. Hence, we specified 18 *f* and *p* parameters, an intercept and slope coefficient for each of one-way (*f*1, *f*2, *f*3) and two-way *f* parameters (*f*12, *f*13, *f*23); and an intercept as well as the slope parameters for each of the detection models. The model equation below implies for 2-way *f* parameters:

$$f_{{{12}}} = \, \upbeta_{{{7 } + }} \upbeta_{{8}} \left( {{\text{Covariate}}} \right),{\text{ p }} = \, \upbeta_{{{13 } + }} \upbeta_{{{14}}} \left( {{\text{covariate}}} \right)$$

$$f_{{{13}}} = \, \upbeta_{{{9 } + }} \upbeta_{{{1}0}} \left( {{\text{Covariate}}} \right),{\text{ p }} = \, \upbeta_{{{15}}} + \, \upbeta_{{{16}}} \left( {{\text{covariate}}} \right)$$

$$f_{{{23}}} = \, \upbeta_{{{11 } + }} \upbeta_{{{12}}} \left( {{\text{Covariate}}} \right),{\text{ p }} = \, \upbeta_{{{17}}} + \, \upbeta_{{{18}}} \left( {{\text{covariate}}} \right)$$

A total of 38 models were run to test the influence of environmental variables on occupancy and detection probability of species-specific (*f*1, *f*2, *f*3) and pair-wise interaction of the three ungulate species. The best-supported model was identified by selecting the model with the lowest AICc value and highest model weights^66^, where higher model weights indicate a better fit of the model to the data. Second-Order Information Criterion (AICc)^67^ values were used to rank the occupancy models, and all the models whose ΔAICc < 2 were considered as equivalent models. The Akaike weight represents the ratio of ΔAICc values for the whole set of candidate models, providing a strength of evidence for each model. The sign of the logistic coefficient of each variable (positive or negative) was used to determine the direction of influence of the variables on the occupancy and detection probability of the three ungulate species.

### Activity pattern

We have compared activity patterns among species to see how overlapping patterns may relate to the competition using the package “Overlap” in R (R Development Core Team). The time and date printed on the photographs have been used to determine the daily activity pattern of individual species^68^. We used a Daily Activity Index (DAI) of half an hour duration to examine the daily activity. The coefficient of overlap is denoted by “Dhat1” values, ranging between zero (no overlap) and 1.0 (complete overlap).
